# Supplementary figures and images for: Establishment of a well-characterized SARS-CoV-2 lentiviral pseudovirus neutralization assay using 293T cells with stable expression of ACE2 and TMPRSS2
Source: PLoS One. 2021 Mar 10;16(3):e0248348. doi: 10.1371/journal.pone.0248348 (PMC7946320; doi:10.1371/journal.pone.0248348)

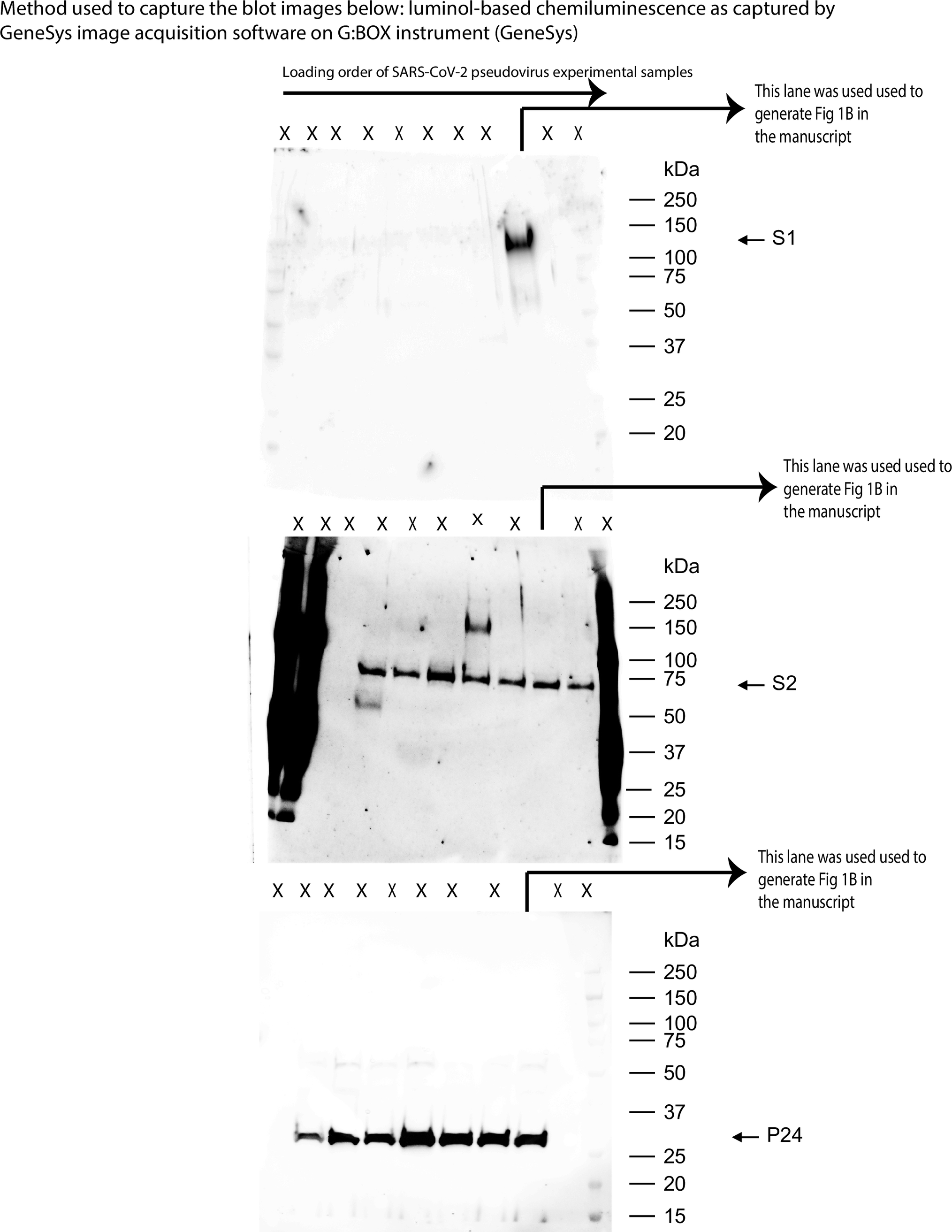

Supplement: S1 Raw images — (TIF) [file pone.0248348.s001.tif]
